# Knowledge, attitudes, and help-seeking behaviour for mental illness in a Christian community

**DOI:** 10.4102/sajpsychiatry.v29i0.2139

**Published:** 2023-11-07

**Authors:** Nomthandazo Hlongwane, Vidette Juby

**Affiliations:** 1 Discipline of Psychiatry, School of Clinical Medicine, University of KwaZulu-Natal, Durban, South Africa

**Keywords:** mental health, knowledge, attitudes, Christian community, help seeking

## Abstract

**Background:**

Christian beliefs have a role in conceptualising mental illness, which determines help-seeking behaviour and treatment choices. The topic of mental illness is controversial in many Christian circles and is often avoided because of the beliefs and teachings stemming from the Christian faith. Inadequate and inaccurate knowledge about mental illness and its causes negatively impacts the attitudes towards mental illness, the mentally ill, and ultimately help-seeking behaviour.

**Aim:**

This study aimed to explore knowledge, attitudes, and help-seeking behaviour for mental illness in a Christian community.

**Setting:**

A descriptive, quantitative cross-sectional survey was conducted, comprising 300 congregants from a Pentecostal Christian charismatic church – Assemblies of God in KwaZulu-Natal.

**Methods:**

A socio-demographic tool was used to capture the relevant social, demographic, and religious information. Existing self-administered questionnaires were used to collect information in four areas: Mental Health Knowledge Schedule, the Reported and Intended Behaviour Scale, Community Attitudes Towards Mentally Ill scale and the Dimensions of Religiosity Scale to determine the degree of religiosity.

**Results:**

The population studied demonstrates high levels of mental health knowledge and a tolerant attitude towards persons who have mental illness. There is a high preference for professional treatment.

**Conclusion:**

The high knowledge in this Christian community is associated with a reduction in stigma and a positive attitude towards mental illness. If mental health awareness is encouraged, it helps to encourage positive help-seeking practices tolerance, and treatment outcomes.

**Contribution:**

This study highlights levels of knowledge and its influence on help-seeking practices and stigma in a Christian community.

## Introduction

Mental illness is a major contributor to the global burden of disease.^1^ Results from the first nationally representative study of psychiatric morbidity in South Africa indicate that approximately 30% of adults will experience a psychiatric disorder in their lifetime. The majority of South Africans are likely to have a direct or indirect encounter with mental illness in their lifetime.^1^

Spirituality and religion play a significant role in mental health. Its significance is evident in individual consultations with mental healthcare users as some tend to rely on their faith to help them to recover and overcome their distress.^2,3^ However, there is a risk that the mental healthcare user will not comply with medical treatment modalities if his or her religious beliefs conflict with the medical approach. Spirituality has a significant influence on patients’ attitudes and practices contributing to mental illness and recovery.^2,3,4^

Spiritual beliefs and practices can have a positive influence on mental health associated with better mental health outcomes.^5^ Involvement in a spiritual society results in enhanced social support and social interaction and can shape how one relates with others.^3,5^ Spiritual involvement also provides guidelines for lifestyle choices including healthy living choices. During trial and suffering, people derive meaning and purpose from their spiritual beliefs.

Although spiritual beliefs can relieve distress, they may be inadequate when symptoms are severe and professional intervention is needed.^6^ This was the finding of a systematic review on spiritual health by Harold Koenig which showed that, when a person’s distress had progressed beyond a certain severity, spiritual beliefs may not be sufficient to bring about relief. In a state where there is an inability to mobilise adequate spiritual resources, professional mental health intervention is required to return the afflicted to the state of being able to draw comfort from their spiritual belief. It can be difficult to distinguish between mental and emotional disorders when they are entangled with spiritual believe. This poses a challenge in making a distinction between religious believing a cause of distress (liability) as opposed to it being a means of help (a resource).^6,7^

Studies show that conservative Christians, including charismatics, are more likely to attribute the cause of mental illness to spiritual factors and believe that faith is the most effective treatment option and thus encourages spiritual-based treatment modalities.^8^ Such teaching is associated with the attitude that mental illness does not exist, and once a person becomes a Christian, they are immune to mental illness.^8,9^

Charismatic Christianity is a fast-growing religious denomination that has special views that speak to miraculous acts as a solution to worldly problems including disease.^10,11^ According to the Bible, Jesus instructed his 12 disciples to heal the sick as he did when he was on earth (Bible Luke Chapter 9). Miraculous acts have since been attributed to the Holy Spirit. The modern Pentecostal religious movement began when a Methodist minister sought answers on how the miracles recorded in the Bible New Testament came about. This movement is associated with unabashed emotionality, belief in personal contact with the Holy Spirit as manifested by speaking in strange tongues, and healing through spiritual means.^7,10^

Religious beliefs are further influenced by ethnicity and culture in predicting attitudes and interpretation of mental illness.^7,12^ Even within a specified denomination, there are differences in beliefs and attitudes about mental illness, which are attributed to the specific ethnic group even when the general teachings are not different.^13^ The approach to addressing mental illness is often influenced by religious beliefs among African Christian and Muslim communities. This choice is guided by the belief in mental illness having a spiritual origin over organic cause.^6,13,14^ There are varying approaches to addressing mental health issues among Christians.^3,12,15,16^ Conservative Christians may turn to religious support, often citing sin and a lack of self-control as the root of mental illness. This perspective can sometimes create a reluctance to seek professional help, but many still find comfort in their faith. Meanwhile, liberal Christians tend to prioritise seeking assistance from trained health professionals who share their religious beliefs, to avoid being misunderstood. To better address mental health issues, promoting greater awareness and education about mental illness can help reduce the stigma associated with seeking professional help, while also encouraging individuals to seek the support they need.^12,15^

This study aimed to explore the knowledge, attitudes, and help-seeking behaviour for mental illness among congregants in a Pentecostal Christian charismatic church in KwaZulu-Natal.

## Research methods and design

This was a descriptive, quantitative cross-sectional survey of congregants from a Pentecostal Christian charismatic church – Assemblies of God in KwaZulu-Natal.

The church members were approached with the permission of the church leadership using an existing communication method (WhatsApp Group) as well as directly.

As per the inclusion criteria, all members and leadership (Church Pastors and committee members) of the Assemblies of God church aged 18 years and above were eligible to participate. Furthermore, inclusion in the study required the participant to have the ability to give consent and have English literacy. The study was conducted with permission solely from KwaZulu-Natal branches, which resulted in the exclusion of church members residing outside the province.

The survey link is linked to a Google forms database https://docs.google.com/forms/d/e/1FAIpQLSer1BN4Is-2e3BSxSQj-QmTEJF8bukjGSvUQW4FxsHVhbVI9w/), which was forwarded to the participants via WhatsApp and email. Participants completed the survey online, and the results were entered into a database.

The sample size was based on the statistical parameters, of which the proportion was assumed to be 0.8 and the confidence interval 1.96; the required sample size for the study would be approximately 246. To make allowance for attrition, a 10% increase was made. It was assumed that this sample size would reduce the type 1 and type 2 errors as well as known and unknown confounding effects. Thus, power (1-beta) (the % chance of detecting difference) of the study was set at 80%. Given the aforesaid, the minimum sample size was set at 300 participants for this study.

The survey included a socio-demographic tool that was used to capture the relevant social, demographic, and religious history details. The following self-administered questionnaires were used to collect information on knowledge, attitude, and behaviour: Mental Health Knowledge Schedule (MAKS), The Reported and Intended Behaviour Scale (RIBS), Community Attitudes Towards Mentally Ill scale (CAMI) and the Dimensions of Religiosity scale (Appendix 1). It should be observed that the questionnaires used have yet to be formally validated in South Africa. However, it is important to mention that permission to utilise these tools through the Indigo network, which was obtained upon registration, was secured.

### Mental Health Knowledge Schedule

The MAKS scale rates 12 items on a 5-point Likert scale (agree strongly, agree slightly, neither agree nor disagree, disagree slightly, disagree strongly). This scale covers stigma-related mental health knowledge as well as respondents’ ability to recognise various conditions as mental illnesses. This tool has been used in several studies in the ‘Time to Change’ anti-stigma campaign in the UK and is a reliable and validated tool (internal consistency, Cronbach’s alpha = 0.65; overall test-retest reliability, Lin’s concordance statistic = 0.71).^17^

### The Reported and Intended Behaviour Scale

This tool measures behaviour with regard to mental health about stigma. The RIBS is a measure of mental health stigma-related behaviour. It comprises eight items scored on a cardinal scale of 1–5. It is an existing tool that is validated, was developed in London, and can be used in conjunction with the MAKS tools. There is a strong consensus on the validity and comprehensibility as rated by users and international experts in the field of stigma research. Overall test-retest reliability of the RIBS was 0.75 (Lin’s concordance statistic) and the overall internal consistency among items was 0.85 Cronbach’s alpha.^18^

### Community Attitudes towards Mentally Ill Scale

The CAMI scale consists of 27 items, which respondents are asked to rate on a five-point Likert scale from 1 (strongly agree) to 5 (strongly disagree). This scale evaluates the participants’ attitude towards individuals with mental illness. Taylor and Dear’s research yielded satisfactory alpha coefficients (AU, α = 0.68; BE, α = 0.76; SR, α = 0.80; CMHI, α = 0.88) for the internal consistency of the subscales. This scale evaluates the participants’ attitude towards individuals with mental illness. Taylor and Dear’s research yielded satisfactory alpha coefficients (AU, α = 0.68; BE, α = 0.76; SR, α = 0.80; CMHI, α = 0.88) for the internal consistency of the subscales.^19^

### The Dimensions of Religiosity Scale

This is a 20-item tool self-report measure of religious pre-occupation, guidance, conviction, and emotional involvement. The 20-item Dimensions of Religiosity Scale (DR Scale) is scored on a 5-point Likert scale, ranging from strongly agree (5) to strongly disagree (1), with the scoring reversed for negatively worded items. The total scale was also highly internally reliable with a Cronbach’s alpha of 0.95 (Cronbach, 1951). This tool has been validated and used in many research studies. It is easy to complete and takes approximately 5 min to complete.^20^

The total scores of CAMI, MAKS and RIBS scales, 25th, 50th and 75th percentile were considered as cut-off points for a low, medium and high scores (Table 1).

**TABLE 1:** Calculation of cut off scores values for measurement tools.

Scale	High score cut off	Medium score cut off	Low score cut off	Maximum possible score
MAKS	48	44	40	60
CAMI	96	81	71	135
RIBS	17	14	8	20

MAKS, Mental Health Knowledge Schedule; CAMI, Community Attitudes towards Mentally Ill scale; RIBS, Reported and Intended Behaviour Scale.

### Ethical considerations

Ethical approval for the study was obtained from the Biomedical Research Ethics Committee of the University of KwaZulu-Natal (approval BREC/00002281/2021). Informed consent was obtained from all participants. The commencement of the online survey encompasses an introductory page that furnishes comprehensive particulars about the survey, comprising pertinent information concerning consent and ethical standards.

### Statistical analysis

Data were captured on Excel spreadsheets and analysed using IBM SPSS version 27. Descriptive analysis statistics were performed for percentages, means and their corresponding standard deviation in data analysis. To determine if demographic variables can predict outcomes, logistic regression analysis was performed. Statistical significance was set at *p* < 0.05.

## Results

### Socio-demographic characteristics of the participants

A total of 300 respondents met criteria and completed the questionnaires, of which 185 (61.7%) were female and 115 (38.3%) were male. The largest group of respondents were between 18 years and 20 years of age (*n* = 108, 36%), whereas only 10% of the group were over the age of 50 years. Most of the participants (*n* = 163, 54%) had completed high school while only one participant had no formal education. More than half of the respondents had been attending their current church for 5 years or less. The Sociodemographic data of respondents can be seen in Table 2.

**TABLE 2:** Sociodemographic data of respondents (*N* = 300).

Variables	Category response	*n*	%
Gender	Male	115	38.3
	Female	185	61.7
Ethnicity	Black people	279	93.0
	White people	4	1.3
	Indian people	2	0.7
	Mixed race people	15	5.0
Age (years)	18–20	108	36.0
	21–30	64	21.3
	31–40	59	19.7
	41–50	39	13.0
	50+	30	10.0
Level of education	Primary	6	2.0
	Secondary	28	9.3
	Matric	163	54.3
	Tertiary	102	34.0
	No school	1	0.3
Length of time as a Christian	1 year	38	12.7
	2–5 years	118	39.3
	6–10 years	41	13.7
	11–20 years	39	13.0
	21+ years	63	21.0
	Missing	1	0.3
Length of time at current church	1 year or less	42	14.0
	2–5 years	121	40.3
	6–10 years	50	16.7
	11–20 years	35	11.7
	21+ years	52	17.3
Who are participants likely to consult?	Lay Christian counsellor	46	15.3
	Church Pastor	76	25.3
	Christian professional	62	20.7
	Hospital or clinic	108	36.0
	Traditional healer	7	2.3
	Missing	1	0.3

### Mental Health Knowledge Schedule

High knowledge scores were recorded for 141 (47%) participants and 110 (37%) respondents scored in the moderate knowledge range. There were 49 (16%) participants with low-level mental health knowledge and attitude. There were high levels of agreement that schizophrenia, bipolar and depression are types of mental illness. Schizophrenia and bipolar disorder were correctly identified as a mental illness by 76.3% and 71.7% of respondents, respectively. Depression and drug addiction were less often recognised as mental illnesses (61% and 58% of respondents, respectively).

### Community Attitudes towards Mentally Ill scale

Higher CAMI scores indicate less stigma. Medium scores of public stigma towards mental illness were most common in respondents: 141 (46.8%) had a medium score while 96 (31.6%) had more favourable attitude (high score). Half of the male respondents (*n* = 58; 50%) scored in and above the 75th percentile indicating favourable attitudes towards mental illness and most females, that is, 96 (52%) had a medium attitude response. Across the ethnic groups, most scores were in the 50th percentile with intermediate attitude. The tabulated results of CAMI can be seen in Table 3.

**TABLE 3:** Community attitudes towards mentally ill (*N* = 300).

Statement	Results									
	Agree strongly	%	Agree slightly	%	Neutral	%	Disagree strongly	%	Disagree slightly	%
1. One of the main causes of mental illness is a lack of self-discipline and willpower.	124	41.3	50	16.7	1	0.3	125	41.7	0	0
2. There is something about people with mental illness that makes it easy to tell them from normal people.	115	38.3	119	39.7	0	0	66	22.0	0	0
3. As soon as a person shows signs of mental disturbance, he should be hospitalised.	181	60.3	45	15.0	0	0	74	24.7	0	0
4. Mental illness is an illness like any other	108	36.0	55	18.3	0	0	137	45.7	0	0
5. Less emphasis should be placed on protecting the public from people with mental illness.	129	43.0	103	34.3	0	0	68	22.7	0	0
6. Mental hospitals are an outdated means of treating people with mental illness.	127	42.3	57	19.0	0	0	116	38.7	0	0
7. Virtually anyone can become mentally ill.	104	34.7	55	18.3	0	0	141	47.0	0	0
8. People with mental illness have for too long been the subject of ridicule.	106	35.3	127	42.3	0	0	67	22.3	0	0
9. We need to adopt a far more tolerant attitude towards people with mental illness in our society.	127	42.3	114	38.0	0	0	58	19.3	0	0
10. We have a responsibility to provide the best possible care for people with mental illness.	134	44.7	105	35.0	0	0	61	20.3	0	0
11. People with mental illness don’t deserve our sympathy.	156	52.0	87	29.0	0	0	57	19.0	0	0
12. People with mental illness are a burden on society.	143	47.7	95	31.7	0	0	62	20.7	0	0
13. Increased spending on mental health services is a waste of money.	137	45.7	58	19.3	0	0	105	35.0	0	0
14. There are sufficient existing services for people with mental illness.	109	36.3	111	37.0	0	0	80	26.6	0	0
15. People with mental illness should not be given any responsibility.	104	34.7	104	34.7	0	0	92	30.6	0	0
16. A woman would be foolish to marry a man who has suffered from mental illness, even though he seems fully recovered.	174	58.0	44	14.7	0	0	79	26.3	0	0
17. I would not want to live next door to someone who has been mentally ill.	125	41.7	96	32.0	0	0	79	26.3	0	0
18. Anyone with a history of mental problems should be excluded from taking public office.	141	47.0	91	30.3	0	0	68	22.7	0	0
19. No one has the right to exclude people with mental illness from their neighbourhood.	138	46.0	56	18.7	0	0	106	35.3	0	0
20. People with mental illness are far less of a danger than most people suppose.	138	46.0	85	28.3	0	0	77	25.7	0	0
21. Most women who were once patients in a mental hospital can be trusted as babysitters.	154	51.3	67	22.3	0	0	79	26.3	0	0
22. The best therapy for many people with mental illness is to be part of a normal community.	128	42.7	104	34.7	0	0	68	22.7	0	0
23. As far as possible, mental health services should be provided through community-based table facilities.	125	41.7	66	22.0	0	0	109	36.3	0	0
24. Residents have nothing to fear from people coming into their neighbourhood to obtain mental health services.	123	41.0	98	32.7	0	0	79	26.3	0	0
25. It is frightening to think of people with mental problems living in residential neighbourhoods.	106	35.3	122	40.7	0	0	72	24.0	0	0
26. Locating mental health facilities in a residential area downgrades the neighbourhood.	130	43.3	45	15.0	0	0	125	41.7	0	0
27. People with mental health problems should have the same rights to a job as anyone else.	96	32.0	128	42.7	0	0	76	25.3	0	0

*Source:* Taylor SM, Dear MJ. Scaling community attitudes toward the mentally ill. Schizophr Bull. 1981;7(2):225. https://doi.org/10.1093/schbul/7.2.225

### Reported and Intended Behaviour Scale

Subscale A – indicates previous or past behaviours, with high scores indicating a higher contact with the mentally ill. There were 139 (46%) who scored medium and 95 (31.6%) who scored high indicating a fairly high community exposure to the mentally ill.

The majority of respondents (*n* = 154; 51.3%) reported either currently living with or previously having lived with someone with a mental health problem. However, 63% (*n* = 189) of respondents reported not having worked with someone with a mental illness, and a high number of participants, 186 (62%) reported no history of having neighbours with mental health problems (Table 4a).

**TABLE 4a:** Reported and Intended Behaviour Scale.

Statements in subscale 1: Reported behaviour	Results					
	Yes	%	No	%	Neutral	%
1. Are you currently living with, or have you ever lived with, someone with a mental health problem?	154	51.3	123	41.0	23	7.7
2. Are you currently working with, or have you ever worked with, someone with a mental health problem?	81	27.0	189	63	30	10
3. Do you currently have, or have you ever had, a neighbour with a mental health problem?	93	31.0	186	62.0	21	7.0
4. Do you currently have, or have you ever had, a close friend with a mental health problem?	84	28	193	64.3	23	7.7

*Source:* Mental Health Knowledge Schedule MAKS 10 © 2009 Health Service and Population Research Department, Institute of Psychiatry, King’s College London

#### Subscale B

Subscale B scores reflect positive attitudes towards individuals with mental illness, indicating a willingness to engage with and treat them with kindness. The mean score was 12.47 with a standard deviation of 5.78. However, a small percentage of participants (less than 25%) reported a lack of willingness to have any interaction with those experiencing mental illness (Table 4b).

**TABLE 4b:** Reported and Intended Behaviour Scale.

Statements in intended behaviour subscale 2	Results									
	Agree strongly	%	Agree slightly	%	Neutral	%	Disagree	%	Disagree	%
5. In the future, I would be willing to live with someone with a mental health problem.	60	20	119	39.7	43	14.3	71	23.7	7	2.3
6. In the future, I would be willing to work with someone with a mental health problem	69	23.0	105	35	46	15.3	70	23.3	10	3.3
7. In the future, I would be willing to live near by to someone with a mental health problem	65	21.7	116	38.7	41	13.7	69	23.0	9	3.0
8. In the future, I would be willing to continue a relationship with a friend who developed a mental health problem	70	23.3	116	38.7	37	12.3	68	22.7	9	3.0

*Source:* Mental Health Knowledge Schedule MAKS 10 © 2009 Health Service and Population Research Department, Institute of Psychiatry, King’s College London

### Bivariate analysis

Bivariate analysis with MAKS scores as a dependent variable showed higher scores in males compared with females (45.78 vs 42.53, *p* < 0.001). The highest mean scores were in the 31–40-year age group while the lowest knowledge scores were found in the 50+ age group (43.41 vs 40.64, *p* < 0.001).

Level of education was significantly associated with knowledge levels. Finishing secondary school was associated with higher knowledge (*r* = 0.36, *p* < 0.005). There were 70 (43%) participants with high school qualification who scored in the 50th percentile (moderate knowledge) and 68 (42%) in the 75th percentile (high knowledge) and 54 (52%) participants with tertiary education scored in the 75th percentile (high knowledge). In comparison to other groups, those with only a primary school level of education had the lowest level of knowledge and secondary education respondents had the highest scores (39.5 vs. 44.62). Furthermore, it was found that individuals who have been practising Christianity for 11–20 years displayed the highest level of knowledge. Bivariate analysis of CAMI scores shows that the male gender is associated with higher scores and more favourable attitudes compared with females (92.0 vs 80.7). Stigma is lowest in the 31–40- and 41–50-year age groups and highest in the 18–20 age group (75.9 vs 95.98).

There was no significant correlation between attitude and age or length of time as a Christian.

There was a statistically significant relationship between the level of education and attitude towards mental illness. A level of education beyond high school predicted a favourable attitude (*r* = 0.015 *p* < 0.005). Of the respondents who had completed matric (*n* = 163; 54.3%), 52 (31.5%) had high scores and 64 (39%) had medium scores indicating intermediate attitudes. Of respondents with tertiary education (*n* = 102), 34 (33%) had high scores and 55 (53%) had medium scores.

There is a weak correlation between MAKS and CAMI, with a Pearson correlation of *r* = 0.02, *p* < 0.005.

The male gender was also associated with favourable behaviour compared with female (14.63 vs. 11.15). Favourable intended behaviour was higher in older age groups. The lowest RIBS scores (lower favourable behaviour) were found in the adolescent group and highest in the 50+ and above age group (8.99 vs 15.44). Primary education is associated with less favourable behaviours compared with having tertiary education (8.33 vs. 14.35). Community Attitudes towards Mentally Ill Scale scores were positively correlated with RIBS scores (*r* = 0.594, *p* < 0.05). A favourable community attitude is correlated with a positive intended behaviour for participants.

Logistic regression analysis suggests that the number of years as a Christian was not predictive of the level of knowledge of mental illness and did not predict attitude significantly (*p* = < 0.005, *r* = 0.188); however, respondents’ level of education was significantly predictive of greater knowledge (*p* < 0.005, *r* = 0.361). The tabulated results of the bivariate analysis can be seen in Table 5.

**TABLE 5:** Bivariate analysis of demographic data and the Community Attitudes towards Mentally Ill Scale, Mental Health Knowledge Schedule, Reported and Intended Behaviour Scale scores.

Variable	CAMI		MAKS		RIBS	
	Total	*P*	Total	*P*	Total	*P*
**Gender**	-	< 0.001‡	-	< 0.001‡	-	< 0.001‡
Male	92 ± 19.97	-	45.78 ± 4.32	-	14.65 ± 5.2	-
Female	80.7 ± 14.33	-	42.53 ± 4.65	-	11.15 ± 5.74	-
**Race**	-	0.438	-	0.012‡	-	0.824
Black people	85.06 ± 17.83	-	43.85 ± 4.69	-	12.41 ± 5.92	-
White people	75 ± 5.6	-	40†	-	14.5 ± 1.29	-
Mixed race people	82.13 ± 9.93	-	39.1 ± 5.2	-	12.6 ± 5.78	-
Indian people	97.5 ± 24.75	-	48†	-	15 ± 7.07	-
**Age (years)**	-	< 0.001‡	-	< 0.001‡	-	< 0.001‡
18–20	75.9 ± 12.17	-	43.41 ± 4.04	-	8.99 ± 5.93	-
21–30	85.63 ± 16.01	-	42.92 ± 5.06	-	13.75 ± 5.12	-
31–40	95.98 ± 19.27	-	45.3 ± 4.61	-	14.41 ± 4.94	-
41–50	91.75 ± 19.28	-	45.15 ± 4.85	-	14.91 ± 4.63	-
50+	85 ± 11.34	-	40.64 ± 6.01	-	15.44	-
**Level of education**	-	0.032‡	-	< 0.001‡	-	0.306‡
Primary	71.8 ± 14.24	-	39.5 ± 3.54	-	8.33 ± 3.2	-
Secondary	86.78 ± 14.27	-	44.62 ± 5.29	-	13.17 ± 5.39	-
Matric	82.71 ± 1.57	-	43.86 ± 4.5	-	11.44 ± 6.17	-
Tertiary	88.13 ± 17.44	-	43.06 ± 5.17	-	14.35 ± 4.68	-
**Duration ofChristian belief(years)**		< 0.001‡	-	< 0.001‡	-	< 0.001‡
≤ 1	76.82 ± 11.71	-	43.32 ± 6.63	-	14.76 ± 4.83	-
2–5	76.86 ± 11.8	-	43.04 ± 3.57	-	9.13 ± 5.75	-
6–10	87.05 ± 16.12	-	43.8 ± 4.87	-	13.08 ± 5.57	-
11–20	96.64 ± 19.61	-	45.78 ± 4.63	-	14.73 ± 4.59	-
21+	96.68 ± 18.44	-	43.64 ± 5.37	-	16.02 ± 3.13	-

MAKS, Mental Health Knowledge Schedule; CAMI, Community Attitudes towards Mentally Ill Scale; RIBS, Reported and Intended Behaviour Scale.

† , Only one response, therefore no standard deviation calculated;

‡ , Statistically significant values.

### Dimensions of religiosity score

The majority of respondents had high scores above 80%, indicating a high level of religiosity. There is a negative correlation between level of religiosity and attitudes, Pearson correlation (*r* = -0.203, *p* < 0.005). A high level of religiosity is associated with unfavourable attitude towards mental illness. There is a negative correlation between RIBS and religiosity (*r* = -0.175, *p* < 0.005). There is no significant correlation between level of knowledge (MAKS) and level of religiosity.

## Discussion

The results show that there is a moderate to high level of knowledge in this Christian community regarding mental illness. There appears to be a tolerant attitude towards the mentally ill which is correlated with a positive intended behaviour. A high level of knowledge is associated with a reduced stigma.

### Knowledge

This study has shown moderate to high levels of mental health knowledge. These results differ from other African studies, which have shown low levels of mental health knowledge.^21,22^ In the study, factors that were associated with high knowledge were male sex, level of education beyond high school and young age.

The results indicate that being male is associated with higher knowledge. According to this research conducted, it was observed that male participants had a greater level of education beyond matriculation in comparison to their female counterparts. Moreover, the male participants were generally of a younger age, which could have led to their increased exposure to educational campaigns and mental health literacy. There was a similar result in a study by Birkie et al.^22^, which showed that females had poorer knowledge of mental illness compared with males; it is speculated that females could have lower levels of knowledge because they tend to minimise symptoms and underreport.^22,23,24,25^ Mental health literacy rather than gender was the strongest predictor of positive mental health knowledge and attitude in a study by Lee et al.^23^ Older females, however, had lower levels of knowledge and negative mental health attitude.^26^

Having completed secondary level education is associated with better knowledge. This may imply that mental health knowledge is improved by education. This result is in keeping with other studies which show that higher education is associated with higher knowledge.^21,22^ A study evaluating anti-stigma social campaigns in Ghana and Kenya revealed that there was a low level of knowledge of mental illness as baseline, with some improvement after educational campaigns.^21^ This result of high knowledge in this study could be because the majority of respondents in our study have a higher level of education (matric and tertiary education). Studies have speculated that individuals with a low level of education have a less ability to understand health information and they also have limited access to this information.^22^ While a similar study carried out in Ethiopia by Jarso et al. (different region) revealed poor knowledge towards mental illness, the majority of respondents in that study had low level of education with only 27% having tertiary education.^27^

The increase in age was associated with higher levels of knowledge. The highest levels of mental health literacy were observed among individuals aged 31 years – 40 years, followed by those aged 41 years – 50 years. Although previous studies have suggested that adults are generally less skilled at recognising mental illness, this study yielded a different outcome. However, the reason for this discrepancy remains unclear. Recent research suggests that young adults have a tendency to over-identify with depression,^28,29^ potentially because of increased exposure to mental health education through social media campaigns that aim to raise awareness about depression and suicide.^30^ Other studies had similar results where younger age was associated with a low level of knowledge and a low level of stigma related knowledge.^21^ Other studies found no association between age and level of knowledge.^22,30^

### Help seeking attitude

The majority of participants showed a preference for a hospital in help-seeking. Respondents had a moderate to high level of knowledge about mental illness with an inclination to refer to professionals for treatment. This is consistent with previous studies, which demonstrate that high levels of knowledge influence help seeking that favours westernised methods of treatment, but this does not negatively influence the congregant’s attitude towards people who are mentally ill.^15,31,32^

The majority of participants prefer hospitals, clinics or professional help when dealing with mental illness. The difference could be attributed to the higher levels of knowledge, which increases preference for Westernised treatment instead of traditional methods. Based on the research conducted by Hugo et al.,^33^ it appears that a majority of the participants find medication and psychotherapy to be effective in treating mental illness.

In South African studies, low levels of knowledge with high stigmatisation is a common finding and it is observed to be influencing help seeking negatively. According to Hugo et al., the preferred treatment is talk therapy as mental illness is viewed as being stress-related rather than a medical disorder.^33^ According to recent research, it has been found that only a quarter of church members would approach their pastor for assistance with mental health concerns.^11,34^ This suggests that there is a growing recognition of the biological aspects of mental illness and its influence on individuals’ willingness to seek support.

This could be attributed to the beliefs about mental illness in the Christian context. However, the sample in this study is a small representation as there are many Christian followers and different denominations. Considering the representation sample, the results are not a conclusive representation; they can be viewed as an explorative view. As the knowledge of mental illness increases, there is also an increase in favourable attitudes in the community, that is, less stigma.

There were low levels of stigma related to mental health knowledge in our study. Furthermore, there was a high score of benevolence demonstrated towards the mentally ill people and this finding is consistent with previous studies. A study carried out in Ethiopia assessing knowledge and associated factors towards mental illness also showed poor knowledge of mental illness and moderate to high levels of unfavourable attitudes.^22^

### Behaviour and attitudes

Our study shows that respondents have a favourable attitude towards the mentally ill. The high level of knowledge is associated with moderately favourable attitude. This is not in keeping with a study by Jarso et al., which showed that higher knowledge does not predict favourable attitudes. There is no association between good knowledge and negative attitudes.^30^ In a study by Birkie et al., poor knowledge was associated with unfavourable attitude, which is consistent with our findings.^22^ Most survey participants are willing to live with and work with individuals facing mental health challenges and maintain friendships with them. Familiarity and exposure to mental illness is associated with an increase in knowledge and less desire for restrictiveness and distancing from the mentally ill. Similarly, in our study there are favourable attitudes towards those who have mental illness even though there is a negative attitude towards mental illness itself; this can be explained by the high level of exposure to mental illness in this group. A study by Robinson that investigated patterns of change in knowledge, attitudes, and social distancing with people with mental illness over years during an anti-stigma campaign found that women demonstrated a significant decrease in stigma after campaigns, which was associated with increased exposure to and familiarity with people with mental illness.^27^

### Level of religiosity

According to a study conducted by Potts and Henderson,^21^ individuals who identify as Christians exhibited lower levels of knowledge pertaining to mental illness when compared with non-religious populations and other religions. Conversely, those who reported having no religious affiliation displayed a more positive attitude towards mental illness. This study, however, did not find a correlation between level of religiosity and level of knowledge. This finding could be as a result of generally high religiosity in the study participants.

## Conclusion

Through this research, insight into the knowledge, attitudes, and help-seeking behaviours of a particular Christian community was gained. Findings indicate that a considerable number of individuals in this population have been impacted by mental illness, indicating a potentially high incidence rate. The high knowledge in this Christian community is associated with a reduction in stigma and a positive attitude towards mental illness. If mental health awareness is encouraged, it helps to encourage positive help-seeking practices, tolerance, and treatment outcomes.

### Recommendations

There is an importance for mainstreaming mental health in church programme to de-stigmatise mental illness. Active involvement of Christian organisations in providing families and carers of those with mental illness to increase familiarity and involvement will also help in destigmatising the mentally ill. The Church community can help with mental health advocacy and promotion.

### Limitations

The study employed anonymous self-reported questionnaires, which may not fully represent the Christian population. A potential limitation could be neutral response bias, where participants may not feel comfortable expressing their true attitudes and may therefore provide untruthful reporting. In addition, self-report questionnaires could result in a loss of introspective ability and reporting bias. It is worth noting that the instruments used in the study have yet to be validated in the South African context, and the study only included one denominational cluster, which reduces the generalisability of the results. To address this, future studies should consider including other denominations.

## Figures and Tables

**FIGURE 1-A1:**
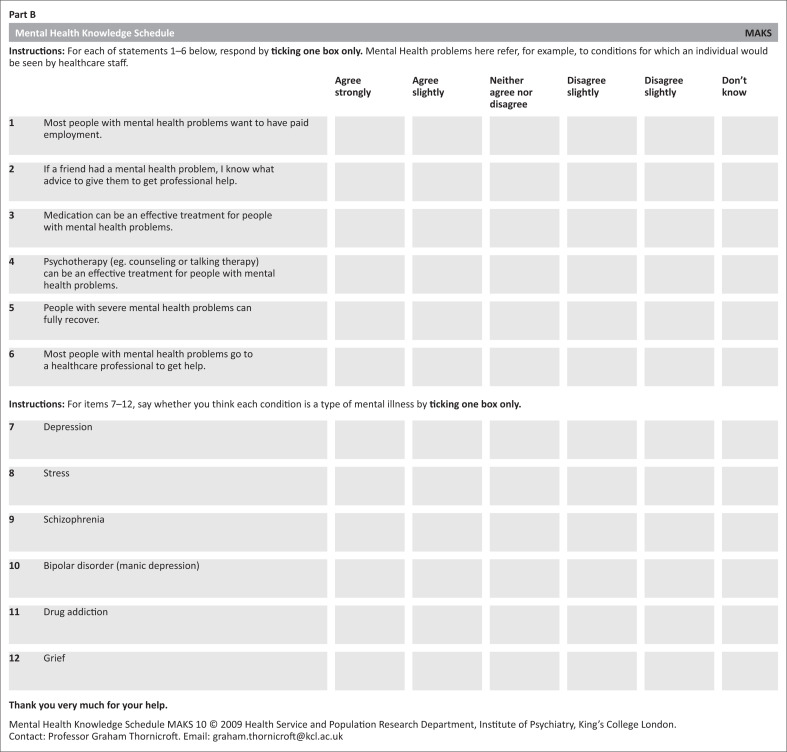
Part B.

**FIGURE 2-A1:**
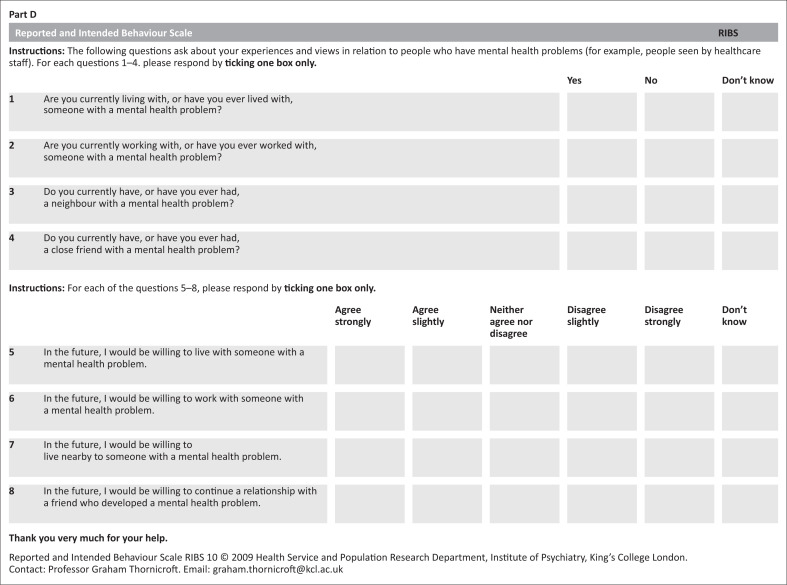
Part D.

**FIGURE 3-A1:**
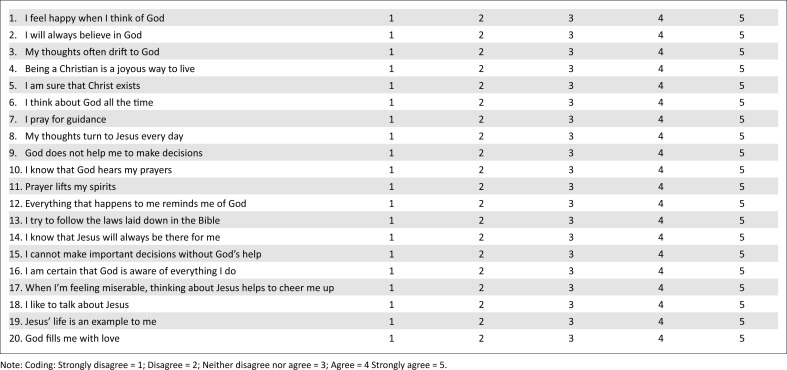
Dimensions of religiosity scale
